# Supplementary material for: Gac Is a Transcriptional Repressor of the Lyme Disease Spirochete’s OspC Virulence-Associated Surface Protein
Source: J Bacteriol. 2023 Mar 15;205(4):e00440-22. doi: 10.1128/jb.00440-22 (PMC10127594; doi:10.1128/jb.00440-22)
Supplement: Supplemental file 1 — Tables S1 and S2 and Fig. S1 and S2. Download jb.00440-22-s0001.pdf, PDF file, 0.4 MB [file jb.00440-22-s0001.pdf]

Gac is a transcriptional repressor of the Lyme disease spirochete's

OspC virulence-associated surface protein

by

Tatiana N. Castro-Padovani, Timothy C. Saylor, Olivia T. Husted, Andrew C. Krusenstjerna,

Nerina Jusufovic, and Brian Stevenson

Supplemental Tables and Figures

Supplemental Table S1. Sequences of synthetic DNAs and oligonucleotide primers used in this study

**DNA name**	**Purpose**	**Sequence (5' to 3')**
5.1	insert to produce pBLS761	GGTACCGCATATTTGGCTTTGCTTATGTCGATTTTAAAATCAAAT TAAGACAATATTTTTCAAATTCTTCAATATTTATTCAAGATATTGA AGAATTTGAAAAAATTATTTTTTCAAATAAAAAATTGAAAAACA AAATTGTTGGACTAATAATTCATAAATAAAAAGGAGGATCC
5.4	insert to produce pBLS764	GGTACCAATTGAAAAACAAAATTGTTGGACTAATAATTCATAAA TAAAAAGGAGGATCC
ospC 1010	PCR B31 *ospC* promoter/operator for production of pBLS760 and EMSA probe, competitors A, D, F, and G	ATATATGGATCCTCCTTTTTATTTATGAATTATTAG
ospC 1011	PCR B31 *ospC* promoter/operator to produce pBLS756	ATATATGGTACCGCATATTTGGCTTTGCTTATG
ospC 1021	PCR Ip89 *ospC* promoter/operator to produce pBLS756	ATATATGGTACCGCATATTTGGCTTTGCTTCTG
ospC 1021	PCR Ip89 *ospC* promoter/operator for DNA affinity and EMSA probes	ATATATGGATCCTCCTTTTTATATAAATTATTAG
flaB F	Annealed to *flaB* R to form *flaB* Competitor	AACAGGCAAAAGGATTTGCCAAAGTCAGAAATT
flaB R	Annealed to *flaB* F to form *flaB* Competitor	AATTTCTGACTTTGGCAAATCCTTTTGCCTGTT
ospAB F	Annealed to *ospAB* R to form *ospAB* Competitor	GTATTAAGTTATATTAATATAAAAGGAGAATATATT
ospAB R	Annealed to *ospAB* F to form *ospAB* Competitor	AATATATTCTCCTTTTATATTAATATAACTTAATAC

CompB1F	Annealed to CompB1R to form competitor A	GCATATTTGGCTTTGCTTATGTCGATTTTAAAATCAAATTAAGAC AATATTTTTCAAATT
CompB1R	Annealed to CompB1F to form competitor A	TTAAACTTTTTATAACAGAATTAAACTAAAATTTTAGCTGTATTC GTTTCGGTTTATACG
CompB2F	Annealed to CompB2R to form competitor B	AATCAAATTAAGACAATATTTTTCAAATTCTTCAATATTTATTCAA GATATTGAAGAAT
CompB2R	Annealed to CompB2F to form competitor B	TAAGAAGTTATAGAACTTATTTATAACTTCTTAAACTTTTTATAAC AGAATTAAACTAA
761 AF	PCR *ospC* competitor C	GCATATTTGGCTTTGCTTATG
761 DF2	PCR *ospC* competitor E	TTATTCAAGATATTGAAGAATTTGAAA
761 ER3	PCR *ospC* competitors C and E	TCCAACAATTTTGTTTTTCAA
M13 Forward	PCR *ospC* and lp17 telomere probes and competitors	GTAAAACGACGGCCAG
M13 Reverse	PCR *ospC* and lp17 telomere probes and competitors	CAGGAAACAGCTATGAC
TL16g	PCR lp17 telomere region, for cloning	AGACTAATAAAATAATGAATA
TL16h	PCR lp17 telomere region, for cloning	GTATTTTGACTCAAAACTTTA
gyrA-F	Q-RT-PCR of *gyrA* 5’ end	GTAGCCATTCCAACAGCAATTC
gyrA-R	Q-RT-PCR of *gyrA* 5’ end	CGATTCTTTAAGTGAGCCTGAGA
gac-F	Q-RT-PCR of *gyrA* 3’ end	CTTACAACTGCAAGTGGAAAGATAG

gac-R	Q-RT-PCR of *gyrA* 3’ end	ACAATAACACCTCGTGACTTTACT
ospC-F	Q-RT-PCR of *ospC*	TGAAGCGTTGCTGTCATCTAT
ospC-R	Q-RT-PCR of *ospC*	GCATAAGCTCCCGCTAACA
ftsK-F	Q-RT-PCR of *ftsK*	GACCTTCTGATGAGCCAATGT
ftsK-R	Q-RT-PCR of *ftsK*	GCTGCTCTGTTGTAACCTATCT

Supplemental Table S2. Relative changes in OspC protein levels in analyses of *B. burgdorferi* NGR, CKO-1, and CKO-1 (pBLS820), as shown in Figure 5. The immunoblots were imaged with a ChemiDoc MP (Bio-Rad, CA), and densities analyzed with background compensation using Image-Lab software (Bio-Rad). **A.** relative differences between strains at the same temperature. For example, in western 1, CKO-1 produced 23x more OspC than did NGR at 23°C. **B.** relative changes of the same strain at 23°C vs. 35°C. For example, in western 1, NGR produced 32x more OspC after shift to 34°C.

A.

**Western**	**Strain**	**Temperature**	
		**23°C**	**35°C**
**1**	**CKO-1 vs NGR**	23	1.4
	**CKO-1 (pBLS820) vs NGR**	14	1.1
	**CKO-1 vs CKO-1 (pBLS820)**	1.6	1.2
**2**	**CKO-1 vs NGR**	17	0.92
	**CKO-1 (pBLS820) vs NGR**	2.5	0.22
	**CKO-1 vs CKO-1 (pBLS820)**	6.9	4.3
**3**	**CKO-1 vs NGR**	6.4	1.1
	**CKO-1 (pBLS820) vs NGR**	1.5	0.69
	**CKO-1 vs CKO-1 (pBLS820)**	4.2	1.2

B.

**Western**	**Strain**	**Change when Shifted from 23°C to 35°C**
**1**	**NGR**	32
	**CKO-1**	2.0
	**CKO-1 (pBLS820)**	2.6
**2**	**NGR**	72
	**CKO-1**	3.9
	**CKO-1 (pBLS820)**	6.2
**3**	**NGR**	57
	**CKO-1**	10
	**CKO-1 (pBLS820)**	26

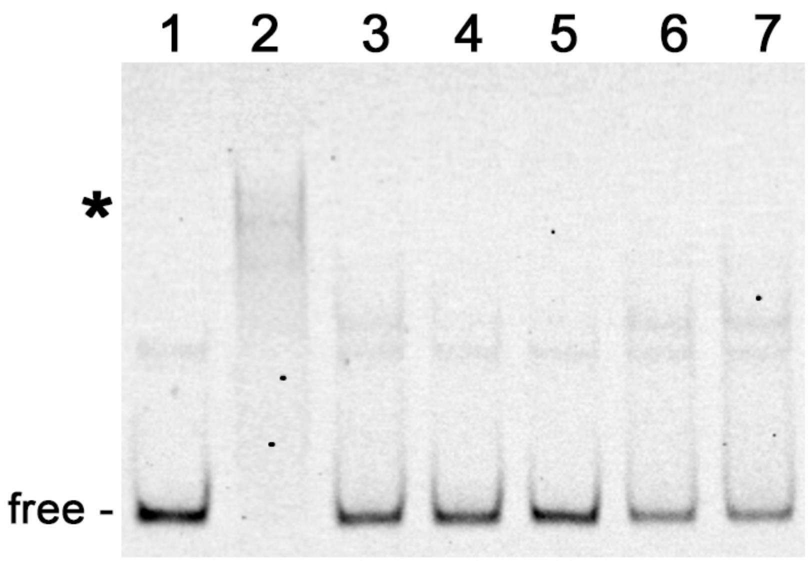

Supplemental Figure S1. Representative EMSA of purified recombinant Gac with labeled B31 *ospC* operator/promoter DNA and unlabeled competitors. Shifted DNAs are indicated with asterisks. Unoccupied DNAs are labeled "free". Labeled B31 operator/promoter (10 nM) without (lane 1) and with 25 nM Gac (lanes 2-7). Lane 2: no added competitor. Lane 3: plus 10x excess unlabeled B31 *ospC* operator/promoter. Lane 4: plus 25x excess unlabeled B31 *ospC* operator/promoter. Lane 5: plus 25x unlabeled pCR2.1 amplicon. Lane 6: plus 25x excess unlabeled *flaB* DNA. Lane 7: plus 25x unlabeled *ospAB* DNA.

-35 -10

CCAGTAAGGATTATTAATATTATAAGAGAAGAAACTATTAATTTAGGTTTGAAATTTGGCGATGAACGTCGAACTAAAATAATTTATGATG

RBS Met Met

AGGAGGTTTTAAAAACTAGT**ATG**TCGGATTTA**ATG**CAAAAAGAAAATATTGTTGTTATGCTTACAAAGAAAGGTTTCCTTAAAAGACTT
TCACAAAATGAGTATAAATTGCAAGGTACGGGAGGAAAAGGACTAAGTTCGTTTGATCTAAATGATGGAGATGAGATTGTTATTGCTTT
GTGTGTCAATACTCATGATTATTTATTTATGATTTCAAATGAAGGAAAGCTTTATTTAATCAATGCTTATGAAATAAAAGATTCTTCAAGAG
CTTCAAAAGGTCAGAATATTAGTGAGCTTATTAATTTAGGAGATCAAGAAGAAATATTAACTATTAAGAATAGTAAAGATTTAACTGATG
ATGCTTATTTATTGCTTACAACTGCAAGTGGAAAGATAGCTAGATTCGAATCTACAGATTTTAAAGCAGTAAAGTCACGAGGTGTTATTG
TTATTAAACTGAATGATAAAGATTTTGTTACAAGTGCAGAGATTGTTTTTAAGGATGAAAAAGTAATTTGTCTTTCTAAAAAGGGTAGTG
CATTTATATTTAATTCAAGGGATGTTAGGCTTACTAATAGAGGTACCCAAGGTGTTTGTGGAATGAAATTAAAAGAAGGTGATTTGTTTG
TTAAAGTTTTATCGGTTAAAGAAAATCCTTATCTTTTGATTGTTTCTGAAAATGGGTATGGAAAAAGGTTAAACATGTCTAAAATATCTG
AGCTTAAAAGAGGAGCCACTGGTTATACTAGTTATAAAAAATCTGATAAAAAAGCGGGTAGTGTTGTTGATGCTATAGCAGTTTCAGAG
GATGATGAAATCTTGCTTGTAAGTAAACGTTCAAAAGCTTTAAGAACAGTAGCTGGAAAAGTATCTGAACAAGGCAAAGATGCTAGAG
GAATTCAAGTATTATTTCTTGATAATGACAGCTTGGTTTCTGTTTCAAAATTTATTAAA**TAA**

Stop

Supplemental Figure S2. Sequence of the insert of pBLS820 that encodes Gac protein. Putative -10 and -35 promoter elements and the ribosome binding site (RBS) are indicated in **purple**. The *gac* ORF is preceded by two in-frame methionine codons, indicated in **green**. The *gac* termination codon is indicated in **red**.
